# *Helicobacter pylori* Eradication Primary Care First‐Line Prescriptions: Data From 200,000 Patients in a Real‐World Cohort

**DOI:** 10.1111/hel.70122

**Published:** 2026-04-01

**Authors:** Encarnación Fernández‐Antón, Olga P. Nyssen, Gabriela Alonso‐Martínez, Pablo Parra, Miguel Gil, Javier P. Gisbert, Francisco J. de Abajo

**Affiliations:** ^1^ Department of Biomedical Sciences (Pharmacology), School of Medicine and Health Sciences University of Alcalá (IRYCIS) Alcalá de Henares Spain; ^2^ Teófilo Hernando Foundation Madrid Spain; ^3^ Department of Gastroenterology Hospital Universitario de La Princesa, Instituto de Investigación Sanitaria Princesa (IIS‐Princesa), Universidad Autónoma de Madrid (UAM), and Centro de Investigación Biomédica en Red de Enfermedades Hepáticas y Digestivas (CIBERehd) Madrid Spain; ^4^ BIFAP Unit, Division of Pharmacoepidemiology and Pharmacovigilance, Spanish Medicines Agency Hospital Universitario de La Princesa, Instituto de Investigación Sanitaria Princesa (IIS‐Princesa) Universidad Autónoma de Madrid (UAM), and Centro de Investigación Biomédica en Red de Enfermedades Hepáticas y Digestivas (CIBERehd) Madrid Spain; ^5^ Clinical Pharmacology Unit, University Hospital Príncipe de Asturias Alcalá de Henares Spain

**Keywords:** bismuth, eradication treatment, *H. pylori*, primary‐specialized care

## Abstract

**Background:**

*Helicobacter pylori* is a common infection primarily managed in primary care. Assessing real‐world practices and guideline adherence is crucial for treatment optimisation. The study aims to assess current *H. pylori* management strategies using data from BIFAP, a Spanish primary care database.

**Materials and Methods:**

Cohort study including patients aged ≥ 18 with recorded *H. pylori* infection (2003–2023) and corresponding treatment prescriptions. Infection cases were identified using ICD‐9/10 and SNOMED‐CT codes. Treatment patterns were based on Spanish and European guidelines. First‐line treatment prescriptions were compared between primary (BIFAP) and specialized (European Registry, Hp‐EuReg) care.

**Results:**

A total of 211,972 *H. pylori‐*infected subjects were identified. Over the study period (20 years), the predominant first‐line treatments were: bismuth quadruple therapy including a proton‐pump inhibitor (PPI) plus a single capsule containing bismuth‐tetracycline‐metronidazole (36%); PPI + clarithromycin‐amoxicillin (30%); and PPI + clarithromycin‐amoxicillin‐metronidazole (26%). Single‐capsule bismuth quadruple therapy was the most common in patients aged 18–64 and those with obesity, chronic kidney disease, or smokers, while PPI + clarithromycin‐amoxicillin was more common in those aged ≥ 65 or with peptic ulcers. Since 2013, PPI + clarithromycin‐amoxicillin use by general practitioners and gastroenterologists decreased, though it remained above 10% in primary care at study end. PPI + clarithromycin‐amoxicillin‐metronidazole increased since 2015, with higher use in specialized care (40%) vs. primary care (30%). In 2023, single‐capsule bismuth quadruple therapy was the most prescribed regimen in both settings, accounting for ~60% of prescriptions.

**Conclusions:**

Primary care *H. pylori* treatments are varied, with single‐capsule bismuth quadruple therapy most prescribed. Guidelines are followed, but adoption is slower in primary than in specialized care.

**Abbreviations**
AEG‐REDCap: Asociación Española de gastroenterología (Spanish Association of Gastroenterology). Research Electronic Data Capture
BIFAP: Base de Datos de Investigación Farmacoepidemiológica en el Ámbito Público (Public Domain Pharmacoepidemiological Research Database)
EHMSG: European Helicobacter and Microbiota Study Group
EHR: Electronic Health Record
Hp‐EuReg: European Registry on the Management of *Helicobacter pylori* Infection
ICD: International Classification of Diseases
PPI: Proton Pump Inhibitor
PPI+A+L: Triple therapy with PPI, amoxicillin, levofloxacin
PPI+A+L+B: Quadruple therapy with PPI, amoxicillin, levofloxacin, bismuth
PPI+A+L+M: Quadruple therapy with PPI, amoxicillin, levofloxacin, metronidazole
PPI+A+M: Triple therapy with PPI, amoxicillin, metronidazole
PPI+A+M+B: Quadruple therapy with PPI, amoxicillin, metronidazole, bismuth
PPI+A+R: Triple therapy with PPI, amoxicillin, rifabutin
PPI+A+R+B: Quadruple therapy with PPI, amoxicillin, rifabutin, bismuth
PPI+C+A: Triple therapy with PPI, clarithromycin, amoxicillin
PPI+C+A+M: Quadruple therapy with PPI, clarithromycin, amoxicillin, metronidazole
PPI+C+L: Triple therapy with PPI, clarithromycin, levofloxacin
PPI+C+M: Triple therapy with PPI, clarithromycin, metronidazole
PPI+C+T: Triple therapy with PPI, clarithromycin, tinidazole
PPI+M+D+B: Quadruple therapy with PPI, metronidazole, doxycycline, bismuth
PPI+M+Tc: PPI, metronidazole, tetracycline
PPI+M+Tc+B: Quadruple therapy with PPI, metronidazole, tetracycline, bismuth
RB+C+A: Ranitidine‐bismuth, clarithromycin, amoxicillin
RB+M+C: Ranitidine‐bismuth, metronidazole, clarithromycin
RB+Tc+M: Ranitidine‐bismuth, tetracycline, metronidazole
ScBQT: Quadruple therapy with PPI and a single capsule Pylera (ATC A02BD08: metronidazole, tetracycline, bismuth, Laboratoires Juvisé Pharmaceuticals, Villeurbanne, France)
SNOMED‐CT: Systematised Nomenclature of Medicine – Clinical Terms
PPV: Positive Predictive Value

## 1 Introduction

*Helicobacter pylori (H. pylori)* is a gram‐negative bacterium, formally identified in human gastric biopsies by Marshall and Warren in 1982 [1]. This bacterium infects over half of the population worldwide, representing a significant healthcare burden [2]. This infection is the main cause of certain important diseases, including chronic gastritis, peptic ulcer disease, and gastric cancer [3, 4]. Eradicating the infection improves dyspepsia, prevents peptic ulcer recurrence and its complications, and lowers gastric cancer risk. Thus, guidelines recommend treatment for all infected patients [5, 6].

In Spain, *H. pylori* infects approximately 50% of the population and is a major cause of digestive consultations [7]. Though traditionally seen as a digestive disease, its unclear transmission mechanism calls for a public health approach, prioritizing diagnosis and treatment in primary care. In our setting, the *test‐and‐treat* strategy—non‐invasive diagnostic *H. pylori* test and treating the infection if present—is recommended for dyspeptic patients under 55 years old without alarm symptoms [6, 8]. This reduces unnecessary endoscopies and improves the efficiency of dyspepsia management. In fact, currently most *H. pylori* ‐infected patients are diagnosed and prescribed an eradication treatment by family physicians, highlighting the need for well‐designed prevention strategies to ensure effective treatments (i.e., with at least 90% efficacy) [6].

The European Registry on *H. pylori* Management (Hp‐EuReg; https://www.hpeureg.com) is an international, non‐interventionist, multicentre study endorsed by the European Helicobacter and Microbiota Study Group (EHMSG) [9]. It systematically evaluates therapeutic outcomes in patients treated by gastroenterologists across Europe to align clinical practices with guidelines. With currently over 80,000 enrolled patients (one‐third from Spain) and 38 countries since 2013, the registry highlights persistent management inconsistencies despite gradual improvements in prescriptions, adherence and efficacy [10, 11, 12].

However, the Hp‐EuReg includes only cases managed by gastroenterologists and excludes primary care, where most *H. pylori* diagnoses and treatments occur. To address this gap, we analyzed real‐world epidemiological data from the Spanish Database for Pharmacoepidemiological Research in primary care (BIFAP), focusing on patients who received eradication therapy against *H. pylori* infection. Our objective was to evaluate the various treatment combinations prescribed for *H. pylori* infection in routine primary care practice over 20 years (2003–2023).

## 2 Materials and Methods

### 2.1 Source of Data

We performed an epidemiological cohort study extracted from BIFAP (*Base de datos para la Investigación Farmacoepidemiológica en el Ámbito Público*) [13], a healthcare database containing real‐world data of primary care physicians (PCPs) from 12 out of the 17 Spanish regions. The BIFAP database reflects the Spanish population and has been reliably validated by benchmarking against other prominent European databases used in pharmacoepidemiologic research [14, 15, 16]. BIFAP contains data on demographics, medical conditions, and drug prescriptions, including product name, dosage, indication, date, and duration of supply, along with various other information [13]. In this study, the 2023 version of the BIFAP was used, which included 22.5 million electronic health records (EHRs) of patients and an average of 10 years of follow‐up per subject (a total of 240 million person‐years).

### 2.2 Design

This is a cohort epidemiological study of patients aged 18 years or older who had a recorded *H. pylori* infection between January 1, 2003, and June 30, 2023, and received an eradication therapy against *H. pylori* infection. All subjects were followed for at least 6 months (study end date: December 31, 2023).

### 2.3 Cohort Definition

The study cohort comprised subjects with both a recorded *H. pylori* infection and a subsequent specific first‐line treatment plan.

#### 2.3.1 Identification and Validation of *H. pylori* Infection

We built a case‐search algorithm to mine codes from the International Classification of Diseases (ICD‐9 and ICD‐10) and the Systematized Nomenclature of Medicine—Clinical Terms (SNOMED‐CT) along with their linked text descriptors to search for potential cases of *H. pylori* infection. Additionally, keywords were included in the search algorithm to identify more cases and map them to the appropriate SNOMED‐CT codes. Algorithm construction is detailed in Table S1.

The case‐search algorithm was assessed via manual review of 500 randomly selected EHRs by two independent researchers (EFA and GAM). The diagnosis of the PCP and the following supportive information were considered: infection test confirmation (urea breath test, stool antigen test) and its result; specialist reports; associated symptomatology; and an eradication confirmation test and its result. After consensus on 100 EHRs, 50 were independently reviewed, yielding a kappa of 0.85 (*p* < 0.01). All potential cases were categorized as full valid case (diagnosis plus supportive information), diagnosis‐only valid case (when just diagnosis was recorded by the PCP), non‐valid case, or inconclusive case (when ambiguous information was found). Disagreements were settled by a validation committee composed of two gastroenterologists (JPG, OPN), a senior epidemiologist from BIFAP (MG), a pharmacoepidemiologist (FdA), and the two reviewers (EFA and GAM).

Considering all valid cases, the positive predictive value obtained was 95.9% (95% confidence interval (CI): 93.7%–97.5%). The additional exclusion of valid cases with a diagnosis only (*n* = 31) would lead to a positive predictive value of 95.6% (95% CI: 93.2%–97.3%). Therefore, the case‐search algorithm was considered appropriate to be applied to the whole database.

#### 2.3.2 Identification of the Treatment Combinations Used for *H. pylori* Infection

A treatment‐search algorithm was built based on all the treatment guidelines collected from the Spanish and European Consensus Conferences from 1997 to 2022. These patterns were identified through the prescription records of the different ATC codes at the active principle level for the antibiotics or bismuth included in the treatment and prescribed on the same day for each individual patient. For proton pump inhibitors (PPI), a window of up to 65 days was allowed to capture patients undergoing chronic treatment. Several patterns could be identified for the same subject.

### 2.4 Covariables

The following data were included for each patient: age, sex, history of peptic ulcer, smoking status (never smoker, current smoker, and past smoker), obesity (BMI > 30 kg/m^2^), and chronic kidney disease (CKD).

### 2.5 Statistical Analysis

The values of continuous variables were expressed as the median and interquartile range. Categorical variables were described as proportions. Treatment prevalence was calculated as the proportion of subjects with relevant prescriptions among those treated. Prevalence was also expressed by sex strata, age groups, history of peptic ulcer, smoking status (never smoker, current smoker, past smoker, unknown), obesity, and CKD to assess whether these factors influenced the use of different treatment combinations.

We focused on the analysis of the first‐line treatment prescribed to eradicate *H. pylori* infection. The data obtained were compared with the information collected at AEG‐REDCap and generated in the context of the Hp‐EuReg [9, 17, 18].

The statistical analysis was performed using STATA MP 18.

### 2.6 Ethics Statement for Human Participant

All information available in BIFAP undergoes a double pseudonymisation process (first at the local level within the Autonomous Communities, and again upon integration into the BIFAP system) which ensures that individual identification is highly unlikely. In accordance with European and Spanish regulations on data protection and observational studies involving medicinal products, informed consent is not required when data are anonymised or pseudonymised; therefore, no written consent has been obtained from the patients as there is no patient‐identifiable data included. This study was approved by the Research Ethics Committee for Medicinal Products of the Príncipe de Asturias University Hospital and authorised by the BIFAP Scientific Committee.

### 2.7 Patient and Public Involvement

Patients and members of the public were not involved in the design, conduct, reporting, or dissemination of this research.

### 2.8 Role of Funding Source

This study was promoted by the researchers and partially funded by Fundación Teófilo Hernando. This funder had no role in the conduct of the study, writing or decision to submit the paper for publication.

## 3 Results

### 3.1 Cohort Characteristics

A total of 1,251,290 records related to *H. pylori* were retrieved from BIFAP. Among these, 211,972 patients were identified as having a valid diagnosis record and receiving at least one of the possible treatment patterns. The time interval between the record of a diagnostic test for *H. pylori* infection and the prescription of eradication treatment had a median of 14 days, with an interquartile range of 6–31 days.

The median age at diagnosis of the infection was 55 years (interquartile range, 44–66). Compared to men, women represented 65.2% of all subjects with infection and associated treatment. Women showed lower rates of smoking (13.3% among current and past smokers vs. 18.6% in men) and CKD (1.1% vs. 1.7% in men). In contrast, the prevalence of obesity was higher in women than in men (12.0% vs. 9.2%). A history of peptic ulcer was more frequently recorded in men than in women (3.6% vs. 1.6%) (Table 1).

**TABLE 1:** Characteristics of the cohort.

	Total *n* = 211,972	Women *n* = 138,175 (65.2%)	Men *n* = 73,797 (34.8%)
Stratified age, *n* (%):			
18–39	38,540 (18.2)	25,862 (18.7)	12,678 (17.2)
40–64	113,494 (53.5)	73,260 (53.0)	40,234 (54.5)
65–84	54,242 (25.6)	35,375 (25.6)	18,867 (25.6)
≥ 85	5696 (2.7)	3678 (2.7)	2018 (2.7)
Obesity, *n* (%):	23,375 (11.0)	16,604 (12.0)	6771 (9.2)
History of peptic ulcer, *n* (%):	4894 (2.3)	2229 (1.6)	2665 (3.6)
CKD, *n* (%):	2822 (1.3)	1566 (1.1)	1256 (1.7)
Smoking, *n* (%):			
Non‐smoker	1077 (0.5)	713 (0.5)	364 (0.5)
Current smoker	28,718 (13.6)	16,822 (12.2)	11,896 (16.1)
Past smoker	3400 (1.6)	1575 (1.1)	1825 (2.5)
Missing	178,777 (84.3)	119,065 (86.2)	59,712 (80.9)

Abbreviation: CKD, Chronic kidney disease.

### 3.2 First‐Line Treatment Prescriptions

Throughout the study period, the most frequent first‐line treatment combinations were quadruple therapy with proton pump inhibitor (PPI) and a single capsule Pylera (Laboratoires Juvisé Pharmaceuticals, Villeurbanne, France) (ScBQT) at 36.0%, PPI + C (clarithromycin) + A (amoxicillin) at 30.0%, and the combination PPI + C + A + M (metronidazole) at 26%. Younger patients in the 18–64 years age group more often received ScBQT (37.5%) and PPI + C + A + M (27.6%). Elderly patients (aged between 65 to ≥ 85 years old) were more likely to receive triple therapy PPI + C + A (35.8%) (Table 2). However, when we evaluated more recent data from 2023, the combination ScBQT is by far the leading combination in all age groups (from 55.5% to 58.2%), with the non‐bismuth quadruple combination being the second highest (from 25.2% to 29.0%) and triple therapy PPI + C + A the third highest (from 10.9% to 12.1%) (Table S2). The distribution of the different treatment combinations was similar when stratified by sex (Table S3).

**TABLE 2:** Description of first‐line treatment combinations by age group.

Treatment combinations, *n* (% of column)	Total *n* = 211,972	18–39 years *n* = 38,540 (18.2)	40–64 years *n* = 113,494 (53.5)	65–84 years *n* = 54,242 (25.6)	≥ 85 years *n* = 5696 (2.7)
ScBQT	76,351 (36.0)	15,379 (39.9)	41,672 (36.7)	18,081 (33.3)	1219 (21.4)
PPI + C + A	64,869 (30.6)	9516 (24.7)	33,871 (29.9)	18,633 (34.4)	2849 (50.0)
PPI + C + A + M	55,954 (26.4)	11,585 (30.1)	30,408 (26.8)	12,965 (23.9)	996 (17.5)
PPI + C + M	4626 (2.2)	618 (1.6)	2281 (2.0)	1490 (2.8)	237 (4.2)
PPI + A + L	4649 (2.2)	557 (1.4)	2429 (2.1)	1479 (2.7)	184 (3.2)
PPI + A + M	2301 (1.1)	450 (1.2)	1219 (1.1)	566 (1.0)	66 (1.2)
PPI + A + L + B	1091 (0.5)	127 (0.3)	415 (0.4)	210 (0.4)	15 (0.3)
PPI + C + L	767 (0.4)	123 (0.3)	534 (0.5)	372 (0.7)	62 (1.1)
PPI + M + D + B	700 (0.3)	103 (0.3)	352 (0.3)	231 (0.4)	14 (0.3)
PPI + A + L + M	213 (0.1)	42 (0.1)	86 (0.1)	52 (0.1)	4 (0.8)
PPI + M + Tc + B	184 (0.1)	17 (< 0.1)	97 (0.1)	84 (0.2)	15 (0.3)
PPI + A + M + B	121 (0.1)	14 (< 0.1)	34 (< 0.1)	10 (< 0.1)	4 (0.8)
PPI + M + Tc	62 (< 0.1)	4 (< 0.1)	51 (< 0.1)	44 (0.1)	22 (0.4)
PPI + A + R	32 (< 0.1)	2 (< 0.1)	16 (< 0.1)	12 (< 0.1)	2 (< 0.1)
PPI + C + T	31 (< 0.1)	2 (< 0.1)	17 (< 0.1)	7 (< 0.1)	5 (0.1)
RB + M + C	8 (< 0.1)	1 (< 0.1)	0 (0.0)	1 (< 0.1)	0 (0.00)
PPI + A + R + B	6 (< 0.1)	0 (0.0)	6 (< 0.1)	1 (< 0.1)	1 (< 0.1)
RB + C + A	5 (< 0.1)	0 (0.0)	2 (< 0.1)	3 (< 0.1)	0 (0.0)
RB + Tc + M	2 (< 0.1)	0 (< 0.1)	4 (< 0.1)	1 (< 0.1)	1 (< 0.1)

Abbreviations: A, amoxicillin; B, bismuth; C, clarithromycin; D, doxycycline; L, levofloxacin; M, metronidazole; PPI, proton pump inhibitor; R, rifabutin; ScBQT, single capsule Pylera (containing metronidazole, tetracycline and bismuth); T, tinidazole; Tc, tetracycline.

Over the study period, patients with obesity, CKD, and who were current smokers showed higher use of ScBQT, while those with a history of peptic ulcer were more frequently treated with PPI + C + A (Table 3).

**TABLE 3:** Description of first‐line treatment combinations by comorbidity.

Treatment combinations, *n* (% of column)	First line (Obesity) *n* = 23,375	First line (Peptic ulcer) *n* = 4894	First line (CKD) *n* = 2822	First line (Current smoker) *n* = 28,718
ScBQT	8234 (35.2)	1373 (28.1)	1016 (36.0)	9984 (34.8)
PPI + C + A	6823 (29.2)	1693 (34.6)	744 (26.4)	8835 (30.8)
PPI + C + A + M	6455 (27.6)	1269 (25.9)	811 (28.7)	7854 (27.3)
PPI + C + M	651 (2.8)	207 (4.2)	92 (3.3)	695 (2.4)
PPI + A + L	515 (2.2)	176 (3.6)	62 (2.2)	610 (2.1)
PPI + A + M	265 (1.1)	64 (1.3)	39 (1.4)	292 (1.0)
PPI + C + L	143 (0.6)	27 (0.6)	25 (0.9)	182 (0.6)
PPI + M + D + B	105 (0.5)	27 (0.6)	12 (0.4)	96 (0.3)
PPI + A + L + B	104 (0.4)	27 (0.6)	12 (0.4)	82 (0.3)
PPI + M + Tc + B	29 (0.1)	9 (0.2)	1 (< 0.1)	33 (0.1)
PPI + A + L + M	22 (0.1)	5 (0.1)	5 (0.2)	18 (0.1)
PPI + M + Tc	14 (0.1)	10 (0.2)	1 (< 0.1)	20 (0.1)
PPI + A + M + B	4 (< 0.1)	2 (0.1)	0 (0.0)	7 (< 0.1)
PPI + C + T	4 (< 0.1)	1 (< 0.1)	0 (0.0)	1 (< 0.1)
PPI + A + R + B	3 (< 0.1)	0 (0.0)	2 (0.1)	1 (< 0.1)
PPI + A + R	3 (< 0.1)	3 (0.1)	0 (0.0)	7 (< 0.1)
RB + Tc + M	1 (< 0.1)	0 (0.0)	0 (0.0)	0 (< 0.1)
RB + M + C	0 (< 0.1)	1 (< 0.1)	0 (0.0)	0 (< 0.1)
RB + C + A	0 (< 0.1)	0 (0.00)	0 (0.0)	1 (< 0.1)

Abbreviations: A, amoxicillin; B, bismuth; C, clarithromycin; D, doxycycline; L, levofloxacin; M, metronidazole; PPI, proton pump inhibitor; R, rifabutin; ScBQT, single capsule Pylera (containing metronidazole, tetracycline and bismuth); T, tinidazole; Tc, tetracycline.

#### 3.2.1 Evolution of First‐Line Treatment in Primary Care Alongside the Recommendations of Spanish and European Consensus Guidelines

**FIGURE 1:**
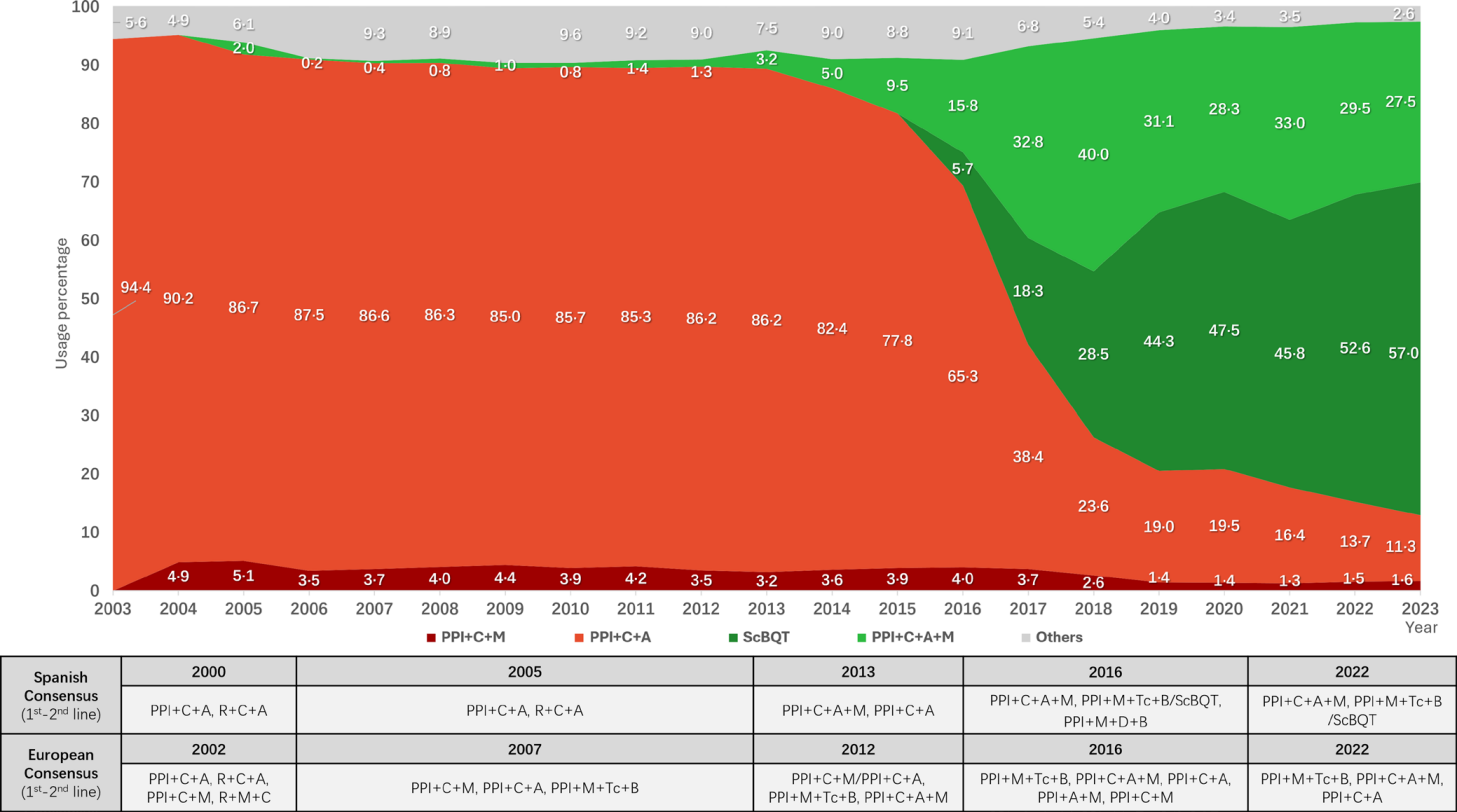
Evolution of first‐line empirical treatment in Spanish primary care from 2004 to 2023 in relation to the recommendations of Spanish and European consensus guidelines. Abbreviations: A, Amoxicillin; B, Bismuth; C, Clarithromycin; D, Doxycycline; L, Levofloxacin; M, Metronidazole; PPI, Proton pump inhibitor; R, Rifabutin; ScBQT, Single capsule Pylera (containing metronidazole, tetracycline and bismuth); T, Tinidazole; Tc, Tetracycline.

Figure 1 shows the trends of first‐line *H. pylori* treatments. From 2003 to 2017, PPI + C + A was the most prescribed combination, aligning with the 2000 [19] and 2005 [20] Spanish and the 2002 [21] European Consensus recommendations. The 2007 [22] European Consensus favored PPI + C + M, and PPI + M + Tc (tetracycline) + B (bismuth) combinations, but such recommendation was not followed and all of them presented use prevalence rates below 5%. After the 2012 [23] European Consensus and the 2013 [24] Spanish Consensus, the prescriptions for PPI + C + A + M combination rose steadily from 3.2% in 2013 to 40.0% in 2018. Starting in 2016, both Spanish [25] and European [26] guidelines recommended bismuth quadruple therapy (PPI, bismuth, tetracycline, metronidazole), and PPI + C + A use strongly declined in favor of PPI + C + A + M and the ScBQT combinations.

#### 3.2.2 Comparison of First‐Line Treatment Between Specialized and Primary Care Settings

**FIGURE 2:**
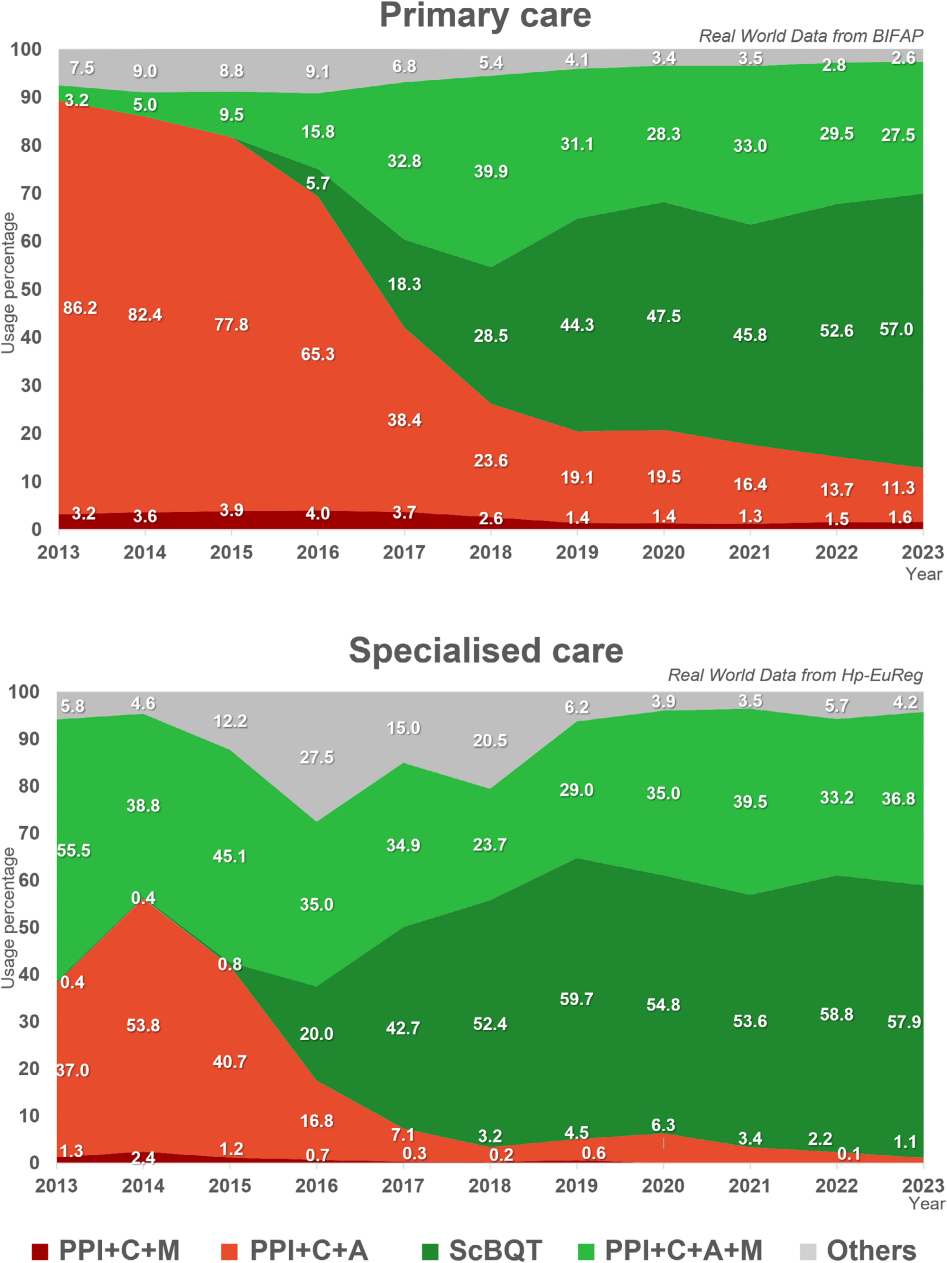
Evolution of eradication treatment prescriptions in primary care as compared to specialized care in Spain. Abbreviations: A, Amoxicillin; BIFAP, *Base de datos para la Investigación Farmacoepidemiológica en el Ámbito Público*; C, Clarithromycin; Hp‐EuReg, European Registry on the Management of *Helicobacter pylori* Infection; M, Metronidazole; PPI, Proton pump inhibitor; ScBQT, Single capsule Pylera (containing metronidazole, tetracycline and bismuth).

Figure 2 displays data from the Hp‐EuReg regarding treatment trends in Spain from 2013 to 2023, alongside comparative data from primary care (BIFAP) for the same period.

Since 2013, the use of PPI + C + A in specialized care has sharply declined from a maximum of 53.8% in 2014 to 1.1% in 2023. This trend was also observed in primary care (from 86.2% in 2013 to 11.3% in 2023). The PPI + C + M combination remained negligible (< 1%) in both settings over the 2013–2023 study period. Conversely, the use prevalence of non‐bismuth quadruple therapy PPI + C + A + M was the highest in specialized care at the start of this period (55.5%), compared to only 3.2% in primary care. Then, while its use remained rather stable until the end of the study period in specialized care, its use grew considerably in primary care, peaking at 39.9% in 2018, when it became the most common regimen, and then stabilized around 30%. The ScBQT (Pylera) has become the most widely prescribed regimen in specialized care since 2017, and in primary care since 2019, accounting for almost 60% of all combinations in both settings.

## 4 Discussion

This study provides the largest and longest real‐world overview to date of *H. pylori* eradication treatment patterns in a primary care population, using a national cohort of over 200,000 patients in Spain. By combining coded diagnoses with prescription data validated through manual review, we captured robust treatment trends over a 20‐year period. Our findings highlight the evolving adherence to European and Spanish guideline‐recommended regimens. Crucially, the data confirmed a progressive shift in *H. pylori* treatment approaches in Spanish primary care. The standard triple therapy (PPI + C + A) was progressively replaced by quadruple regimens, in particular by ScBQT (Pylera). This transition reflects efforts to improve eradication success rates and aligns with current guideline recommendations, although its adoption in primary care has lagged behind that in specialized care.

Over the study period, ScBQT was the most common first‐line treatment overall, particularly among adults less than 65 years old, patients with obesity or CKD, and current smokers. The non‐bismuth quadruple therapy PPI + C + A + M was also common, but less so in the elderly, while PPI + C + A was reported more frequently in this group (particularly among the eldest) possibly due to tolerability or safety concerns with metronidazole or bismuth (of note, however, in 2023 the ScBQT was the combination with the highest prevalence among the elderly at 56%, while triple therapy was only around 12%).

Our data showed a clear shift in first‐line *H. pylori* treatment in Spanish primary care over the last two decades, following evolving guideline recommendations. From 2003 to 2013, PPI + C + A largely predominated, in line with the 2000/2005 Spanish and 2002 European Consensuses [19, 20, 21]. Although the 2007 [22] European Consensus recommended alternatives such as PPI + M + C, PPI + A + M, and PPI + M + Tc + B, their uptake was limited, with PPI + M + C peaking at only 4.4% in 2009.

Following the 2013 Spanish Consensus [24], the use of PPI + M + C + A in primary care increased significantly—from 3.2% to 40% by 2018—aligning with the 2012 [23] European recommendations. From 2016, ScBQT was incorporated into guidelines, and it was gradually adopted. Concurrently, clarithromycin‐based standard triple therapy use declined in favor of bismuth and non‐bismuth quadruple therapies, demonstrating increased adherence to updated clinical guidance and a shift toward regimens with higher eradication efficacy. This evolution occurred several years earlier in specialized care according to data derived from the Hp‐EuReg.

Despite strong evidence discouraging the use of standard triple therapy in regions with high clarithromycin resistance, such as Spain [27], our study shows that its use persisted in primary care with nearly 11% in 2023. In contrast, its use among Spanish gastroenterologists has declined to 1.1%. However, across Europe standard triple therapy is still commonly prescribed, particularly in countries with lower clarithromycin resistance rates (< 15%), often with extended treatment durations or higher acid suppression [10, 28]. As for triple therapy with metronidazole (PPI + C + M), its use in Spain was marginal in primary care and virtually absent in specialized care by the end of the study period. On the other hand, PPI + C + A + M, and especially ScBQT (Pylera), became the dominant first‐line treatment in both settings [29, 30], reflecting broader adherence to updated clinical guidelines [5, 6].

The main limitation of studies using secondary data, like BIFAP, is that the information recorded may not be complete and/or accurate, but prescriptions are issued by the PCPs through the computer, and their recording is almost complete. Although private prescriptions are not included in BIFAP, the National Health System in Spain is universal, primary care is easily accessible, and the cost of medicines is subsidized. Thus, the number of prescriptions issued through the private sector is likely negligible. Despite this limitation, the large sample size—comprising over 200,000 records—combined with the use of real, up‐to‐date clinical practice data from GPs in Spain, provides a robust and comprehensive analysis of the management trends for *H. pylori* infection and current adherence to local clinical guidelines. These findings can be easily applied in routine primary care practice, as the population in BIFAP is representative of the Spanish population treated in that setting.

Therefore, our findings indicate growing adherence toward guideline‐supported therapeutic approaches, especially among patients with comorbidities such as peptic ulcer disease. However, ongoing education and dissemination of updated guidelines among PCPs remains essential. Overall, these results emphasize the importance of national strategies to improve the quality of *H. pylori* management in primary care. Interventions such as expanded access to diagnostic testing, targeted continuing education, and systematized clinical audits successfully implemented in specialist settings should be adapted to the primary care context.

## 5 Conclusions

This large, long‐term, real‐world study provides a comprehensive overview into *H. pylori* eradication practices in Spanish primary care. Although a positive trend toward evidence‐based management is evident, substantial room for improvement remains. Achieving full alignment with current clinical guidelines—particularly regarding optimized treatment selection—remains essential. Structured training, enhanced diagnostic access, and regular auditing are essential to elevate the standard of *H. pylori* management in primary care. While the adoption of guideline‐recommended therapies has been slower and less consistent in primary care, treatment patterns in both care levels have largely converged.

## Author Contributions

Encarnación Fernández‐Antón (EFA) extracted, validated, analyzed, interpreted, and synthesized the data, wrote the first draft, and approved the submitted manuscript. Olga P. Nyssen (OPN) interpreted and synthesized the data, wrote the first draft, and approved the submitted manuscript. Gabriela Alonso‐Martínez (GAM) helped in data validation, critically reviewed the manuscript drafts, and approved the submitted manuscript. Pablo Parra (PP) critically reviewed the manuscript drafts and approved the submitted manuscript. Miguel Gil (MG) contributed to data extraction and data quality control. Javier P. Gisbert (JPG) had the original idea, interpreted and synthesized the data, critically reviewed the manuscript drafts, and approved the submitted manuscript. Francisco J. de Abajo (FDA) had the original idea, interpreted and synthesized the data, critically reviewed the manuscript drafts and approved the submitted manuscript.

## Funding

The *Base de datos para la Investigación Farmacoepidemiológica en el Ámbito Público* (BIFAP) is a public program for independent research financed by the Spanish Agency for Medicines and Medical Devices (AEMPS). This organization had no role in the conduct of the study, writing, or decision to submit the paper for publication. This study was promoted by the researchers and partially funded by Fundación Teófilo Hernando. This funder had no role in the conduct of the study, writing or decision to submit the paper for publication. The Hp‐EuReg project was promoted and funded by the European Helicobacter and Microbiota Study Group (EHMSG; www.helicobacter.org) and received support from the Spanish Association of Gastroenterology (AEG) and the Centro de Investigación Biomédica en Red de Enfermedades Hepáticas y Digestivas (CIBERehd). The Hp‐EuReg was co‐funded by the European Union programme HORIZON (grant agreement number 101095359) and supported by UK Research and Innovation (grant agreement number 10058099). However, views and opinions expressed are those of the author(s) only and do not necessarily reflect those of the European Union or the Health and Digital Executive Agency (HaDEA). Neither the European Union nor the granting authority can be held responsible for them. The Hp‐EuReg was co‐funded by the European Union programme EU4Health (grant agreement number 101101252). The Hp‐EuReg was funded by Diasorin, Juvisé, and Biocodex Microbiota Foundation; however, clinical data were not accessible to the companies, and they were not involved in any stage of the Hp‐EuReg study (design, data collection, statistical analysis, or manuscript writing). This work was supported by Fundación Teófilo Hernando. European Helicobacter and Microbiota Study Group. Spanish Association of Gastroenterology. Centro de Investigación Biomédica en Red de Enfermedades Hepáticas y Digestivas (CIBERehd). European Union programme HORIZON, 101095359. UK Research and Innovation, 10058099. European Union programme EU4Health, 101101252. Diasorin. Juvisé. Biocodex.

## Disclosure

Disclaimer: The results, discussion, and conclusions of this work are only of the authors and do not represent in any way the position on this subject of the AEMPS, or the organizations with which the authors are affiliated.

## Conflicts of Interest

Olga P. Nyssen has served as a speaker or has received research funding from Allergan, Diasorin, Mayoly Spindler, Biocodex, and Juvisé. Javier P. Gisbert has served as speaker, consultant, and advisory member for or has received research funding from Mayoly, Allergan/Abbvie, Diasorin, Richen, Biocodex, and Juvisé. Francisco J. de Abajo has received unrestricted grants from the Institute of Health “Carlos III”, Osteoarthritis Foundation International, Fundación Renal Íñigo Álvarez de Toledo, and the Spanish Agency for Medicines and Medical Devices for other non‐related projects. The remaining authors declare no conflicts of interest. Encarnación Fernández‐Antón (EFA) has access to the data and accepts full responsibility for the conduct of the study.

## Supporting information


**Table S1:** Diagnostic Search Algorithm for *H. pylori* . Codes used according to the corresponding dictionary.
**Table S2:** Description of first‐line treatment combinations by age group in 2023.
**Table S3:** Description of first‐line treatment combinations by sex.

## Data Availability

The main elements of the study protocol were registered in GESTO, part of the Spanish Clinical Studies Registry (REec), prior to data analysis (registration code: 0047–2024‐OBS). The de‐identified, aggregated clinical data generated and analyzed in this study are available upon reasonable request. Due to European Union General Data Protection Regulation (GDPR) constraints and ethical considerations regarding participant confidentiality, raw individual‐level data cannot be shared publicly. However, processed data underlying the study's findings, along with a data dictionary and supporting documentation, may be made available to qualified academic or clinical researchers for non‐commercial and ethically approved purposes. Requests for access to the data must include a detailed research proposal and are subject to review by the BIFAP Data Access Committee. Proposals should be submitted via email to encarnacion.fernande@uah.es. If approved, data will be shared through a secure platform. Additional related documents, including the study protocol, statistical analysis plan, and informed consent form, are also available upon request. Previously published datasets used in this study from the Hp‐EuReg registry can be accessed through the registry's publication page at Hp‐EuReg Publications (www.hpeureg.com). No datasets are included in the Supporting Information or Source Data file associated with this article.
