# Supplementary material for: Helicobacter pylori Eradication Primary Care First‐Line Prescriptions: Data From 200,000 Patients in a Real‐World Cohort
Source: Helicobacter. 2026 Apr 1;31(2):e70122. doi: 10.1111/hel.70122 (PMC13044324; doi:10.1111/hel.70122)
Supplement: Supplementary file 1 — Table S1: Diagnostic Search Algorithm for H. pylori . Codes used according to the corresponding dictionary. Table S2: Description of first‐line treatment combinations by age group in 2023. Table S3: Description of first‐line treatment combinations by sex. [file HEL-31-e70122-s001.docx]

**Supplementary table 1. Diagnostic Search Algorithm for *H. pylori*.
Codes used according to the corresponding dictionary**

| **Code** | **Term** | **Code dictionary** |
| --- | --- | --- |
| 726496002 | Helicobacter pylori resistente a claritromicina | SNOMED |
| 80774000 | Helicobacter pylori | SNOMED |
| 96720031000119104 | úlcera gástrica crónica causada por Helicobacter pylori | SNOMED |
| 307759003 | infección del tracto gastrointestinal por Helicobacter pylori | SNOMED |
| 724528009 | úlcera duodenal causada por Helicobacter pylori | SNOMED |
| 1087081000119102 | hemorragia gástrica causada por Helicobacter pylori | SNOMED |
| 724530006 | úlcera duodenal causada por Helicobacter pylori y agente antiinflamatorio no esteroide | SNOMED |
| 683509791000119109 | úlcera aguda de píloro causada por Helicobacter pylori | SNOMED |
| 724519008 | úlcera gástrica causada por Helicobacter pylori y agente antiinflamatorio no esteroide | SNOMED |
| 1171358000 | gastritis crónica por Helicobacter pylori | SNOMED |
| 221899751000119107 | úlcera pilórica crónica causada por Helicobacter pylori | SNOMED |
| 724525007 | duodenitis causada por Helicobacter pylori | SNOMED |
| 708164002 | gastritis causada por Helicobacter pylori | SNOMED |
| 103691000119106 | úlcera gástrica causada por Helicobacter pylori | SNOMED |
| 89662003 | úlcera pilórica asociada con Helicobacter | SNOMED |
| 724522005 | úlcera anastomótica de estómago causada por Helicobacter pylori | SNOMED |
| 721730009 | infección causada por Helicobacter pylori | SNOMED |
| 182976004 | tratamiento doble contra Helicobacter pylori | SNOMED |
| 182977008 | tratamiento triple contra Helicobacter pylori | SNOMED |
| 736696002 | Helicobacter pylori detectado en materia fecal | SNOMED |
| 444779004 | hallazgo de Helicobacter pylori en prueba de materia fecal | SNOMED |
| 1268771007 | presencia de Helicobacter pylori en materia fecal | SNOMED |
| 168378007 | Helicobacter detectado por prueba de aliento | SNOMED |
| 307369007 | hallazgo relacionado con prueba en el aire espirado para Helicobacter pylori | SNOMED |
| 168385006 | Helicobacter pylori detectado por prueba rápida de ureasa | SNOMED |
| 041.86 | INFECCIÓN POR HELICOBACTER PYLORI [H. PYLORI] | CIE 9 |
| B96.81 | Helicobacter pylori [H. pylori] como causa de enfermedades clasificadas bajo otro concepto | CIE 10 |

**Regular expressions for identifying infection-related keywords**

r'((\bH\b)|(HEL[L]?[IY]{1}C[O]?)|(HEL[L]?[IY]{1}COBACTER))\s*(P[YI]{1}LOR[IY]{1}(?!CO)|P[IY]{1}L(?!ORO|ORICO))'

**Supplementary table 2. Description of first-line treatment combinations by age group in 2023.**

| **Treatment combinations,**  **n (% of column):** | **Total**  **n = 31,201** | **18 – 39 years**  **n = 8,262 (26.5)** | **40 – 64 years**  **n = 16,490 (52.8)** | **65 – 84 years**  **n = 6,132 (19.7)** | **≥ 85 years**  **n = 317 (1.0)** |
| --- | --- | --- | --- | --- | --- |
| ScBQT | 17,791 (57.0) | 4,587 (55.5) | 9,461 (57.4) | 3,567 (58.2) | 176 (55.5) |
| PPI+C+A | 3,527 (11.3) | 945 (11.4) | 1,804 (10.9) | 740 (12.1) | 38 (12.0) |
| PPI+C+A+M | 8,571 (27.5) | 2,395 (29.0) | 4,550 (27.6) | 1,544 (25.2) | 82 (25.9) |
| PPI+C+M | 505 (1.6) | 147 (1.8) | 251 (1.5) | 97 (1.6) | 10 (3.2) |
| PPI+A+L | 178 (0.5) | 36 (0.5) | 92 (0.6) | 47 (0.8) | 3 (0.9) |
| PPI+A+M | 330 (1.1) | 91 (1.1) | 168 (1.0) | 66 (1.0) | 5 (1.6) |
| Other | 299 (1.0) | 61 (0.7) | 164 (1.0) | 71 (1.1) | 3 (0.9) |

A: amoxicillin; C: clarithromycin; L: levofloxacin; M: metronidazole; PPI: proton pump inhibitor; ScBQT: single capsule Pylera^®^ (containing metronidazole, tetracycline and bismuth).

**Supplementary table 3. Description of first-line treatment combinations by sex.**

**Throughout the entire study period**

| **Treatment combinations,**  **n (% of column):** | **Total**  **n = 211,972** | **Women**  **n = 138,175 (65.2)** | **Men**  **n = 73,797 (34.8)** |
| --- | --- | --- | --- |
| ScBQT | 76,351 (36.0) | 50,139 (36.3) | 26,212 (35.5) |
| PPI+C+A | 64,869 (30.6) | 41,812 (30.3) | 23,057 (31.2) |
| PPI+C+A+M | 55,954 (26.4) | 36,192 (26.2) | 19,762 (26.8) |
| PPI+C+M | 4 626 (2.2) | 3 108 (2.2) | 1 518 (2.1) |
| PPI+A+L | 4 649 (2.2) | 3 120 (2.3) | 1 529 (2.1) |
| PPI+A+M | 2 301 (1.1) | 1 557 (1.1) | 744 (1.0) |
| Other | 3 222 (1.5) | 2 247 (1.6) | 975 (1.3) |

A: amoxicillin; C: clarithromycin; L: levofloxacin; M: metronidazole; PPI: proton pump inhibitor; ScBQT: single capsule Pylera^®^ (containing metronidazole, tetracycline and bismuth).

**Throughout 2023**

| **Treatment combinations,**  **n (% of column):** | **Total**  **n = 31,201** | **Women**  **n = 20,470 (65.6)** | **Men**  **n = 10,731 (34.4)** |
| --- | --- | --- | --- |
| ScBQT | 17,791 (57.0) | 11,629 (56.8) | 6,162 (57.4) |
| PPI+C+A | 3,527 (11.3) | 2,355 (11.5) | 1,172 (10.9) |
| PPI+C+A+M | 8,571 (27.5) | 5,569 (27.2) | 3,002 (28.0) |
| PPI+C+M | 505 (1.6) | 341 (1.7) | 164 (1.6) |
| PPI+A+L | 178 (0.5) | 122 (0.6) | 56 (0.5) |
| PPI+A+M | 330 (1.1) | 242 (1.2) | 88 (0.8) |
| Other | 299 (1.0) | 212 (1.0) | 87 (0.8) |

A: amoxicillin; C: clarithromycin; L: levofloxacin; M: metronidazole; PPI: proton pump inhibitor; ScBQT: single capsule Pylera^®^ (containing metronidazole, tetracycline and bismuth).
